# γH2AX, a DNA Double-Strand Break Marker, Correlates with PD-L1 Expression in Smoking-Related Lung Adenocarcinoma

**DOI:** 10.3390/ijms23126679

**Published:** 2022-06-15

**Authors:** Eiko Sakurai, Hisato Ishizawa, Yuka Kiriyama, Ayano Michiba, Yasushi Hoshikawa, Tetsuya Tsukamoto

**Affiliations:** 1 Department of Diagnostic Pathology, Graduate School of Medicine, Fujita Health University, 1-98 Dengakugakubo, Kutsukake-cho, Toyoake 470-1192, Japan; eiko.sakurai@fujita-hu.ac.jp (E.S.); ykiri@fujita-hu.ac.jp (Y.K.); michiba1511@gmail.com (A.M.); 2 Department of Thoracic Surgery, Graduate School of Medicine, Fujita Health University, 1-98 Dengakugakubo, Kutsukake-cho, Toyoake 470-1192, Japan; h-ishi@fujita-hu.ac.jp (H.I.); yasushih@fujita-hu.ac.jp (Y.H.); 3 Department of Diagnostic Pathology, Narita Memorial Hospital, 134, Haneihonmachi, Toyohashi 441-8029, Japan

**Keywords:** lung cancer, adenocarcinoma, squamous cell carcinoma, immune checkpoint inhibitors, programmed death-ligand 1, DNA damage response, γH2AX, smoking, Brinkman index

## Abstract

In recent years, the choice of immune checkpoint inhibitors (ICIs) as a treatment based on high expression of programmed death-ligand 1 (PD-L1) in lung cancers has been increasing in prevalence. The high expression of PD-L1 could be a predictor of ICI efficacy as well as high tumor mutation burden (TMB), which is determined using next-generation sequencing (NGS). However, a great deal of effort is required to perform NGS to determine TMB. The present study focused on γH2AX, a double-strand DNA break marker, and the suspected positive relation between TMB and γH2AX was investigated. We assessed the possibility of γH2AX being an alternative marker of TMB or PD-L1. One hundred formalin-fixed, paraffin-embedded specimens of lung cancer were examined. All of the patients in the study received thoracic surgery, having been diagnosed with lung adenocarcinoma or squamous cell carcinoma. The expressions of γH2AX and PD-L1 (clone: SP142) were evaluated immunohistochemically. Other immunohistochemical indicators, p53 and Ki-67, were also used to estimate the relationships of γH2AX. Positive relationships between γH2AX and PD-L1 were proven, especially in lung adenocarcinoma. Tobacco consumption was associated with higher expression of γH2AX, PD-L1, Ki-67, and p53. In conclusion, the immunoexpression of γH2AX could be a predictor for the adaptation of ICIs as well of as PD-L1 and TMB.

## 1. Introduction

Lung cancer is a common cause of cancer mortality in most developed countries worldwide [1]. Tobacco smoking is one of the most significant causes of cancer development. Many carcinogens found in tobacco have been reported to directly damage and mutate DNA [2]. Although various treatments, including surgery, neoadjuvant chemotherapy, and adjuvant chemotherapy, have been applied to lung cancer patients, the prognosis for these individuals is still poor. In recent years, molecular targeting therapies have been developed and have contributed to a better prognosis for lung cancer.

Tumor mutation burden (TMB) is the total number of somatic missense mutations in the coding genome of tumor tissue or blood samples using whole-exome sequencing. TMB represents the number of mutations per megabase (muts/Mb), and high TMB was defined as being more than 10 muts/Mb [3]. High TMB has been reported to be a biomarker of sensitivity to immune checkpoint inhibitors (ICIs). Although TMB is useful when deciding upon ICI application, the measurement of TMB is costly, labor-intensive, and time-consuming to perform, as it requires next-generation sequencing. Sabari et al. reported that the median turnaround time (TAT) for tissue next-generation sequencing (NGS) was 20 days [4]. PD-L1, which is a ligand of membrane-bound molecule programmed death 1 (PD-1), belongs to the immune checkpoint pathway and is also an indicator for the adaptation of ICIs as well as TMB.

H2AX is a variant of the histone H2A protein, which composes a histone octamer in nucleosomes. Phosphorylated H2AX at the Ser-139 position, called γH2AX, is one of the earliest cellular responses of DNA double-strand breaks. DNA double-strand breaks are induced by ionizing radiation and cytotoxic chemotherapy. The overexpression of γH2AX in cancer cells has been reported in various cancer types [5,6,7,8,9,10]. We hypothesized that the high expression of γH2AX in cancer cells could indicate the high frequency of repairing DNA damage and lead to the high frequency of missense mutations. Therefore, the high expression of γH2AX in cancer might result in high TMB.

Here, we assessed the significance of γH2AX expression as a predictor of the adaptation of ICIs by comparing the immunohistochemical expression of γH2AX with PD-L1. In conclusion, γH2AX, a DNA double-strand break marker, significantly correlated with PD-L1 expression in lung adenocarcinomas, and could be a useful biomarker when deciding upon ICI usage.

## 2. Results

### 2.1. Comparison between Clinicopathological Parameters of Lung Adenocarcinoma and Squamous Cell Carcinoma

Patients were classified as having lung adenocarcinomas or squamous cell carcinomas (Table 1, left panel). There was a greater number of squamous cell carcinomas in men than in women. Adenocarcinomas tend to have lower histological grades than squamous cell carcinomas.

### 2.2. Immunohistochemical Analysis of γH2AX, PD-L1, Ki-67, and p53 in Lung Adenocarcinomas and Squamous Cell Carcinomas

**Figure 1:**
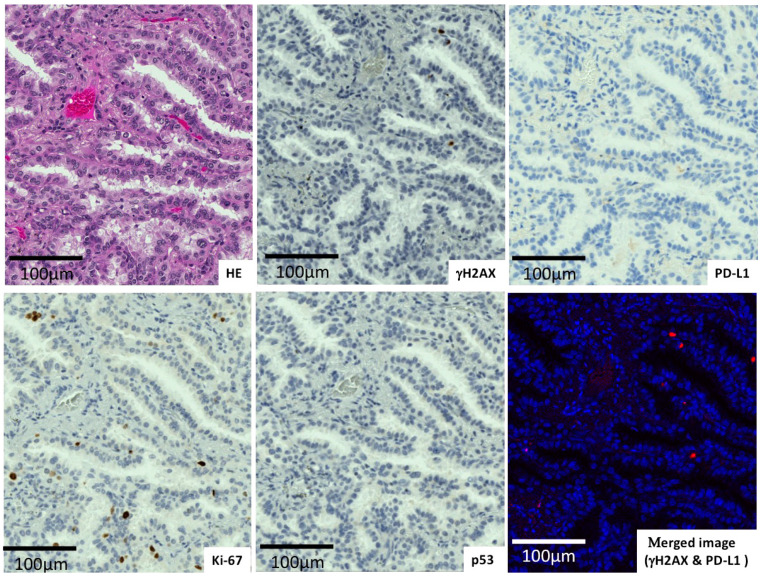
Adenocarcinoma with low γH2AX and PD-L1 levels.

**Figure 2:**
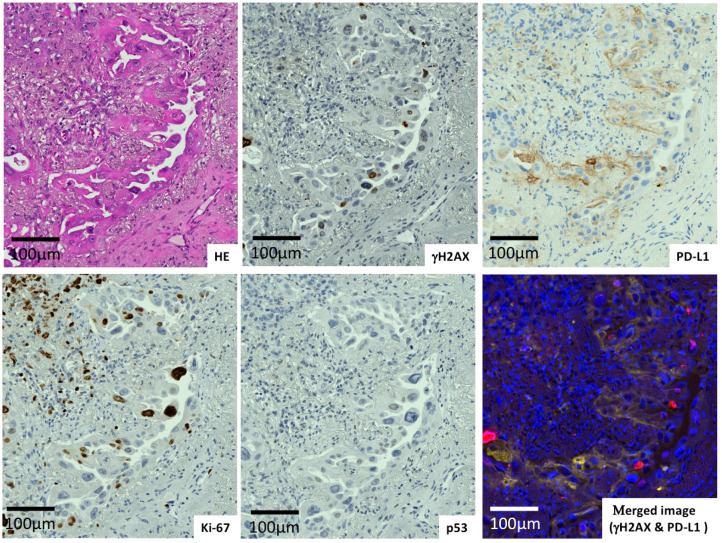
Adenocarcinoma with high γH2AX and PD-L1 levels surrounded by fibrous stroma.

**Figure 3:**
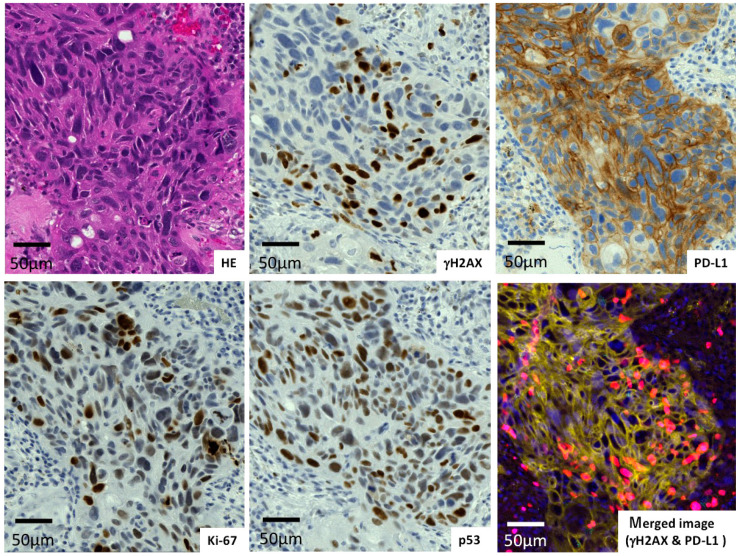
Squamous cell carcinoma with high γH2AX and PD-L1 levels.

The expression of γH2AX, PD-L1, Ki-67, and p53 in lung adenocarcinomas and in lung squamous cell carcinomas was evaluated (Figure 1, Figure 2 and Figure 3). It was shown that one case of adenocarcinoma (Figure 1) possessed low γH2AX and PD-L1 levels with wild-type p53 and a low Ki-67 level. Another adenocarcinoma case (Figure 2) surrounded by fibrotic stroma had higher levels of γH2AX and PD-L1. A case of squamous cell carcinoma was revealed to carry higher levels of those proteins (Figure 3). The merged figure of pseudo-fluorescent images of γH2AX (red) and PD-L1 (yellow) illustrates the similar distribution of those proteins.

### 2.3. Relationship between γH2AX Expression and Clinicopathological Parameters

The expression level of γH2AX was subdivided into low (0–5%) and high (6–100%). Higher γH2AX expression was observed in men compared to women (*p* = 0.0032) and in squamous cell carcinomas compared to adenocarcinomas (*p* < 0.0001). More advanced pT (*p* = 0.0366) and more lymphatic duct invasion were observed in the higher γH2AX group (*p* = 0.0366 and *p* = 0.0119, respectively) (Table 1, right panel).

### 2.4. Difference in Expression of γH2AX, PD-L1, Ki-67, and p53 between Adenocarcinomas and Squamous Cell Carcinomas

**Figure 4:**
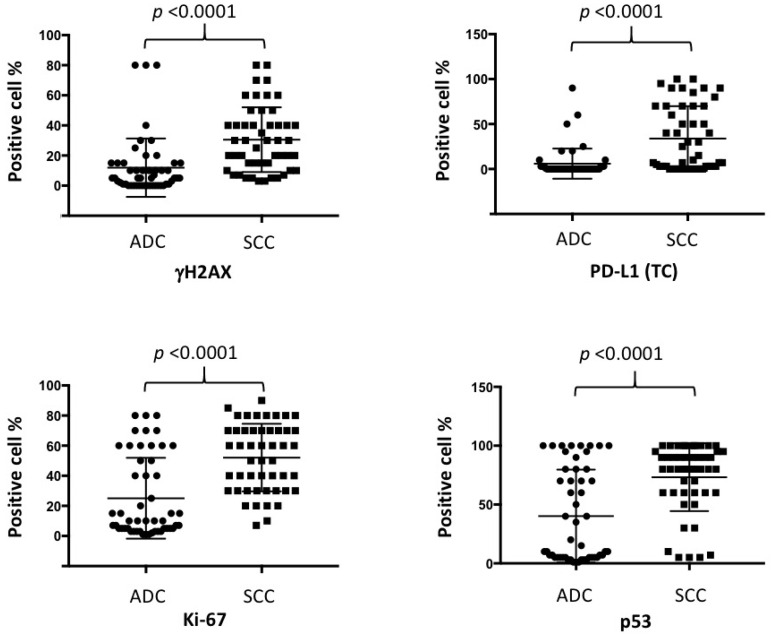
Comparison of immunohistochemical expression of γH2AX, PD-L1(TC), Ki-67, and p53 in adenocarcinomas (ADCs) and squamous cell carcinomas (SCCs).

The expression of γH2AX, PD-L1, Ki-67, and p53 in squamous cell carcinomas was higher than in adenocarcinomas (*p* < 0.0001 in all) (Figure 4).

### 2.5. Relationship between γH2AX Expression and the Immunohistochemical Expression of Other Indicators (PD-L1, Ki-67, and p53)

Cancers with higher γH2AX expression were associated with the higher expression of PD-L1, Ki-67, and p53 (*p* < 0.0001 each) (Table 2).

### 2.6. Correlation between γH2AX and PD-L1 Expression

**Figure 5:**
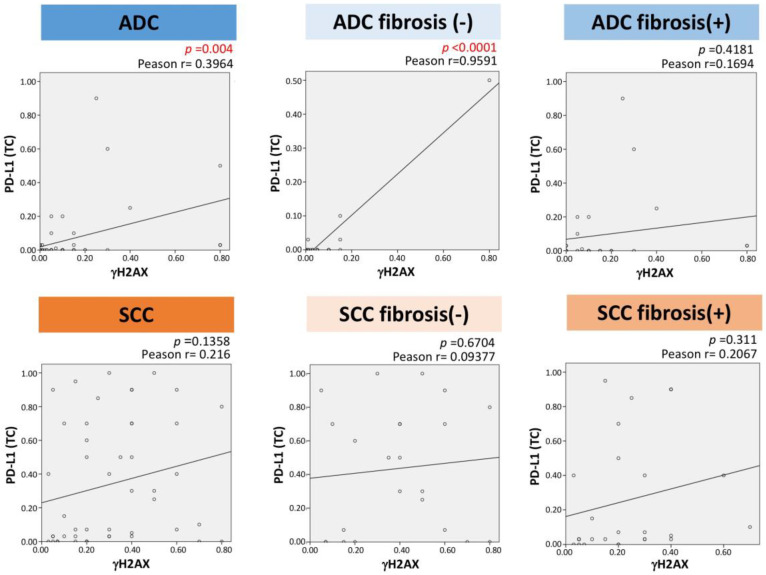
Correlation of γH2AX and PD-L1 immunoexpression.

A positive relationship was found between γH2AX and PD-L1 expression in lung adenocarcinomas (*p* = 0.004, Pearson r = 0.3964), whereas no statistical significance was found in lung squamous cell carcinomas (*p* = 0.1358, Pearson r = 0.216) (Figure 5). Furthermore, a positive relationship was also found in lung adenocarcinomas without surrounding fibrosis, but no statistical significance was found in lung adenocarcinomas with fibrosis.

### 2.7. Relationship between Smoking History (Brinkman Index) and the Expression of γH2AX, PD-L1, Ki-67, and p53

Regarding smoking history (Table 3) and lung cancer histology, most squamous cell carcinoma patients had higher Brinkman index scores compared to those with adenocarcinomas. The group of participants with more extensive smoking histories (Brinkman index ≥ 200) showed higher expressions of γH2AX (*p* < 0.0001), PD-L1(TC score) (*p* = 0.0144), Ki-67 (*p* = 0.018), and p53 (*p* = 0.047). With regard to γH2AX, according to the Brinkman index, there were significant differences in adenocarcinomas (*p* = 0.0349), whereas no significant differences were shown in squamous cell carcinomas (*p* > 0.9999).

### 2.8. Patient Survival

**Figure 6:**
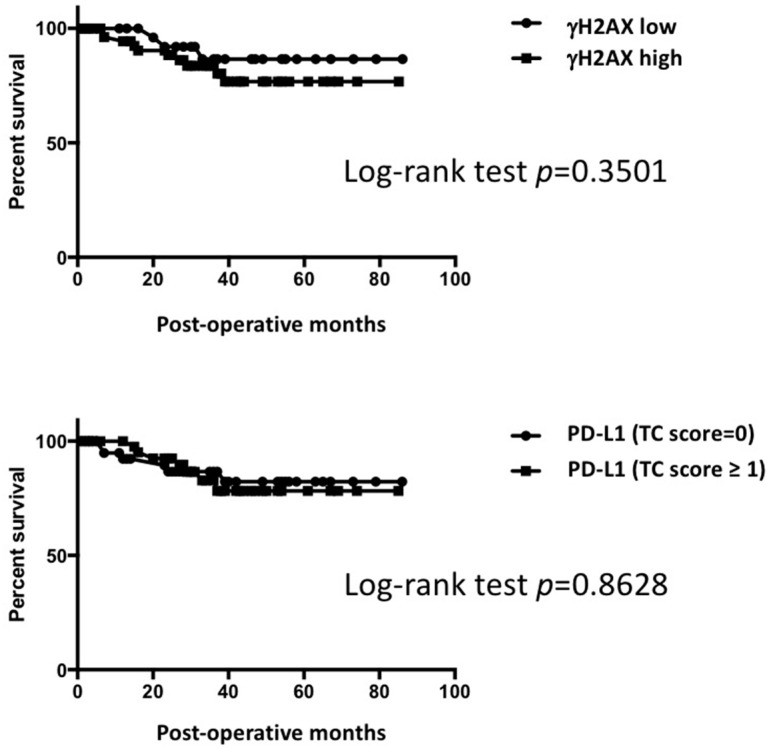
Kaplan–Meier log-rank test showing no statistically significant differences between high and low groups in γH2AX and in PD-L1.

No statistical significance in terms of patient survival was detected in both high/low γH2AX expression and high/low PD-L1 expression (Figure 6, Figures S1 and S2).

## 3. Discussion

In recent years, the prognosis of non-small-cell carcinomas (NSCLCs) with oncogenic driver mutations has improved due to the development of molecularly targeted drugs. However, for NSCLCs without driver mutations, ICIs may be used as a treatment choice. In terms of predicting the effectiveness of ICIs, a high level of the immunoexpression of PD-L1, high TMB, microsatellite instability (MSI), and the tumor infiltration of immune cells indicate that this treatment would be effective. Although TMB is a direct indicator of DNA mutation, its determination requires time-consuming and costly NGS. Therefore, we investigated whether the immunostaining of γH2AX, a DNA double-strand break marker, could be a useful alternative method.

We proved a positive relation between the expression of γH2AX and PD-L1 in lung adenocarcinomas. For subpopulations of adenocarcinomas, positive correlation was confirmed between γH2AX and PD-L1 in adenocarcinoma without fibrosis with statistical significance in contrast to no association in adenocarcinoma with fibrosis [11,12,13]. Celada et al. [11] demonstrated that CD4+ T cells in fibrosis affected high PD-L1 expression and found that higher PD-L1 expression was shown in idiopathic pulmonary fibrosis (IPF) than in the controls. Kronborg-White et al. [12] also demonstrated upregulation of PD-L1 in pulmonary epithelial cells in IPF patients. Kunwar et al. [14] demonstrated that irradiation induced γH2AX expression and subsequent interstitial fibrosis. Taking into account these reports, γH2AX expression could be associated with PD-L1 in interstitial fibrosis. Further analysis may be required to assess the link between γH2AX and PD-L1 expression in adenocarcinoma with fibrosis. In lung squamous cell carcinomas in the present study, no statistical significance was found in correlation between these gene products. Osoegawa et al. reported [15] a positive relationship between γH2AX and PD-L1 expression in lung squamous cell carcinomas, which was determined using PD-L1 clone E1L3N. In the present report, clone SP142, the target therapy of which is atezolizumab, was applied. The outcome of PD-L1 expression might differ depending on the PD-L1 clone used. TMB may be related to tobacco-associated mutagens, but not correlated with PD-L1 expression [16]. Regarding tobacco exposure and PD-L1 expression, Li et al. [17] demonstrated positive correlation. In this study, a positive relationship between tobacco and γH2AX was proved. Depending on previous reports, including our own reports [16,17], the results of investigations between the relationship among PD-L1, γH2AX, and TMB have varied. Thus, this relationship must be examined in a larger number of cases.

The difficulty in evaluating PD-L1 immunohistochemically [18] should also be noted. In most cases, a mixture of high- and low-expression areas of PD-L1 was observed in each tumor because of the heterogeneity. As only one block of FFPE is usually selected for the determination of PD-L1 expression, the highest expression area might not have been included in the selected block. Non-tumor cells, for instance, inflammatory cells and necrotic cells, are also stained by PD-L1. In particular, alveolar macrophages are similar in morphology to cancer cells. It is sometimes difficult to distinguish tumor cells from other cells. The evaluation of PD-L1 by one investigator might not correspond with that of others. While TC (tumor cell) score was used in this study to avoid confusion, IC (immune cell) score was supposed to be included for clinical assessment. Furthermore, the preanalytical processing of tumor samples, including formalin fixation duration, should be properly controlled [19]. On the other hand, it is easier to evaluate γH2AX expression than PD-L1, because inflammatory cells are not stained normally, while necrotic cells are. Furthermore, with regard to γH2AX, it is easier to count positive cells because of its nuclear staining, whereas PD-L1 undergoes membrane staining.

The group of participants with extensive smoking histories (Brinkman index ≥ 200) had high expressions of γH2AX, PD-L1(TC score), Ki-67, and p53. 4-(methylnitrosamino)-1-(3-pyridyl)-1-butanone (NNK), which is nitrosamine derived from tobacco and has been found to cause lung cancer. NNK is metabolized as an ultimate carcinogen by cytochrome 2A6. NNK has been considered to effect p53 mutation and mismatch DNA repair [20,21]. There was a report of the overexpression of Ki-67 or p53 observed in rats who received toxic doses of NNK [22]. The relationship between NNK and γH2AX has also been reported [23]. It was suggested that tobacco-induced carcinogens might induce p53 mutation and the overexpression of γH2AX and Ki-67. The result of high γH2AX expression related to high expression in p53 and Ki-67 could also support this suggestion.

The proper and quick adaptation of ICIs is a major concern, because ICIs might represent a last strategy for the treatment of advanced stages of NSCLCs and NSCLCs without oncogenic driver mutations [24]. Complications regarding ICIs could be fatal in some cases [25], so patients selected for treatment with ICIs must be selected with the utmost caution. Immunohistochemical methods could save time in starting treatment compared to NGS. To make it easy to determine immunohistochemically positive cells, a method must enable uniform evaluation by any judge. The immunostaining of γH2AX is suggested to be a greatly useful method for determining adaptation for ICIs. A larger number of cases needs to be studied. These results warrant further analysis.

In conclusion, a positive relationship between γH2AX with PD-L1 was proven in lung adenocarcinomas. γH2AX could be a predictor of the adaptation of ICIs as an alternative to TMB or PD-L1.

## 4. Materials and Methods

### 4.1. Study Subject

Formalin-fixed, paraffin-embedded (FFPE) specimens from 100 consecutive patients with lung carcinomas were examined. Patients underwent thoracic surgery at Fujita Health University Hospital between February 2015 and August 2020. Their clinicopathological characteristics are shown in Table 1. In total, 80 males and 20 females (age range: 38–87 years; mean: 69.92) were included. Pathological diagnosis was performed according to the fourth edition of the World Health Organization criteria [26] and the eighth edition of the TNM classification of Lung Cancer [27]. In total, 51 patients had lung adenocarcinomas and 49 had squamous cell carcinomas. Lung fibrosis detected with computed tomography was also included to subclassify the carcinomas; 25 and 26 cases involved adenocarcinomas with fibrosis and squamous cell carcinomas with fibrosis, respectively (Table 1).

The Brinkman index, which determines the total amount of tobacco smoking in an individual, was calculated by multiplying the number of cigarettes/day by years of smoking history. Pack-year is 1/20 of the Brinkman index. In the present study, a Brinkman index of 200 was selected as the cut-off value, because the Ministry of Health, Labor, and Welfare of the Japanese Government determined this number or above should be used for the clinical management for tobacco cessation [28].

The use of archived FFPE material of human cases was approved by the Institutional Review Board of Fujita Health University on 23 January 2021 (HM20-326).

### 4.2. Immunohistochemistry

Immunohistochemical investigation was performed using tissue specimens sliced from FFPE. Sections 3 µm thick were deparaffinized in xylene and hydrated in a series of graded ethanol dilutions. Endogenous peroxidase was blocked at room temperature by 0.1% hydrogen peroxide in methanol for 30 min. Heat-induced epitope retrieval was performed in ethylenediaminetetraacetic acid (EDTA) (pH 8.0) and using a pressure cooker for 10 min. The sections were incubated with primary antibodies against γH2AX (rabbit monoclonal, clone 20E3; Cell Signaling Technology, Danvers, MA, 1:1000 dilution), Ki-67 (mouse monoclonal, clone MIB-1; DAKO, Glostrup, Denmark, 1:100 dilution), and p53 (mouse monoclonal, clone DO-7; Novocastra, Newcastle upon Tyne, UK, 1:100 dilution). After being washed, the sections were incubated in goat antimouse/rabbit immunoglobulin labeled with horseradish peroxidase (Histofine Simple Stain MAX-PO, Nichirei, Tokyo) for 30 min at room temperature. Staining was visualized using 3,3′-diaminobenzidine as a chromogen. The slides were then counterstained with hematoxylin. The immunohistochemistry of PD-L1 (mouse monoclonal, clone SP142; Abcam, Cambridge, UK, 1:200 dilution) was performed by using BOND RX Fully Automated Research Stainer (Leica Biosystems, Wetzlar, Germany).

Immunostained slides were evaluated as follows: high γH2AX expression if >5% of tumor cells demonstrated nuclear staining, and high Ki-67 expression if >20% of tumor cells demonstrated nuclear staining. PD-L1 was scored according to the Ventana Optiview PD-L1 (SP142) Judgment guide lung-cancer [29]. PD-L1 TC scores of 0, 1, 2, or 3 were used for our investigation. p53 expression was evaluated by multiplying the intensity (1, 2, or 3, from weak to strong) and proportion (0.0–1.0); a score of more than 1.0 was considered to be abnormal accumulation by mutation.

### 4.3. Merging Multiple Immunohistochemical Images by Utilizing HALO Software (Indica Labs)

Whole-slide images (WSI) of HE and immunohistochemistry were created with a WSI scanner, AxioScan.Z1 (Zeiss, Oberkochen, Germany), using a 20× objective lens. First, immunohistochemical WSI images of γH2AX and PD-L1 were converted to red and yellow pseudo-immunofluorescent images, respectively, and merged views were created using HALO image analysis software (Indica Labs, Albuquerque, New Mexico).

### 4.4. Statistical Analysis

Statistical analysis of the data was performed using Prism 7 (GraphPad, San Diego, CA, USA) and SPSS version 23.0 (IBM, Armonk, NY, USA). The Chi-square test was used to evaluate the association between the molecular expression and the clinicopathological parameters. Pearson’s correlation coefficient (Pearson r) was calculated to evaluate the correlation between γH2AX and PD-L1 expression. The patient survival values were calculated from the day of surgery until death, in months, using the Kaplan–Meier method. *p* values less than 0.05 were considered to be statistically significant.

## Figures and Tables

**Table 1:** Clinicopathological characteristics.

	Histology		*p* Value	γH2AX		*p* Value
	Adenocarcinoma	Squamous Cell Carcinoma		High (6–100%)	Low (0–5%)	
Number of cases	51	49		65	35	
Age						
≥70	28	32	0.3138	41	19	0.402
<70	23	17		24	16	
Sex						
Male	36	44	0.0235	58	22	0.0032
Female	15	5		7	13	
Histology						
Adenocarcinoma	51	0	NA	22	29	<0.0001
Squamous cell carcinoma	0	49		43	6	
Fibrosis						
Fibrosis	25	26	0.6948	36	15	0.2953
Non-fibrosis	26	23		29	20	
Histological grade						
G1–2	48	38	0.0215	55	31	0.7651
G3	3	11		10	4	
pT						
Tis–T1	30	27	0.8401	32	25	0.0366
T2–T4	21	22		33	10	
pStage						
0-I	37	34	0.8117	47	31	0.0779
II-III	13	10		18	4	
pN						
pN0	42	43	0.578	54	31	0.5658
pN1–3	9	6		11	4	
Ly						
Ly0	36	33	0.8296	39	30	0.0119
Ly1	15	16		26	5	
V						
V0	33	32	>0.9999	40	27	0.1256
V1–2	17	17		25	8	
pm						
pm0	49	48	>0.9999	62	35	0.5498
pm1–3	2	1		3	0	
pl						
pl0	41	38	>0.9999	49	31	0.1889
pl1–3	10	10		16	4	

Abbr.: Ly, lymphatic vessel invasion; V, vascular invasion; pm, pulmonary metastasis; pl, pleural invasion.

**Table 2:** Association between γH2AX and the expression of PD-L1, Ki-67, and p53.

	γH2AX		*p* Value
	High (6–100%)	Low (0–5%)	
PD-L1 (TC score)			
0	20	26	<0.0001
1,2,3	45	9	
Ki-67			
Low (0–19%)	11	23	<0.0001
High (20–100%)	54	12	
p53			
Non-mutation	14	23	<0.0001
Mutation	51	12	

**Table 3:** Associations between tobacco (Brinkman index) and histology, and the expression of γH2AX, PD-L1, Ki-67, and p53.

	Brinkman Index			*p* Value
	≥200	<200	Unknown	
Histology				
Adenocarcinoma	33	16	2	<0.0001
Squamous cell carcinoma	44	1	4	
γH2AX				
Total lung cancer				
Low (0–5%)	22	13	0	<0.001
High (6–100%)	55	4	6	
Adenocarcinoma				
Low (0–5%)	16	13	0	0.0349
High (6–100%)	17	3	2	
Squamous cell carcinoma				
Low (0–5%)	6	0	0	>0.9999
High (6–100%)	38	1	4	
PD-L1 (TC score)				
0	32	13	1	0.0144
1,2,3	45	4	5	
Ki-67				
Low (0–19%)	22	12	0	0.0018
High (20–100%)	55	5	6	
p53				
Non-mutation	24	12	2	0.0047
Mutation	53	5	4	

## Data Availability

Data will not be made available, but the data that support the findings of this study are available upon reasonable request to the corresponding author.

## Supplementary Materials

The following supporting information can be downloaded at: https://www.mdpi.com/article/10.3390/ijms23126679/s1.
