# Supplementary material for: γH2AX, a DNA Double-Strand Break Marker, Correlates with PD-L1 Expression in Smoking-Related Lung Adenocarcinoma
Source: Int J Mol Sci. 2022 Jun 15;23(12):6679. doi: 10.3390/ijms23126679 (PMC9223793; doi:10.3390/ijms23126679)
Supplement: Supplementary file 1 [file ijms-23-06679-s001.zip › ijms-1774692-supplementary.pdf]

Supplementary materials

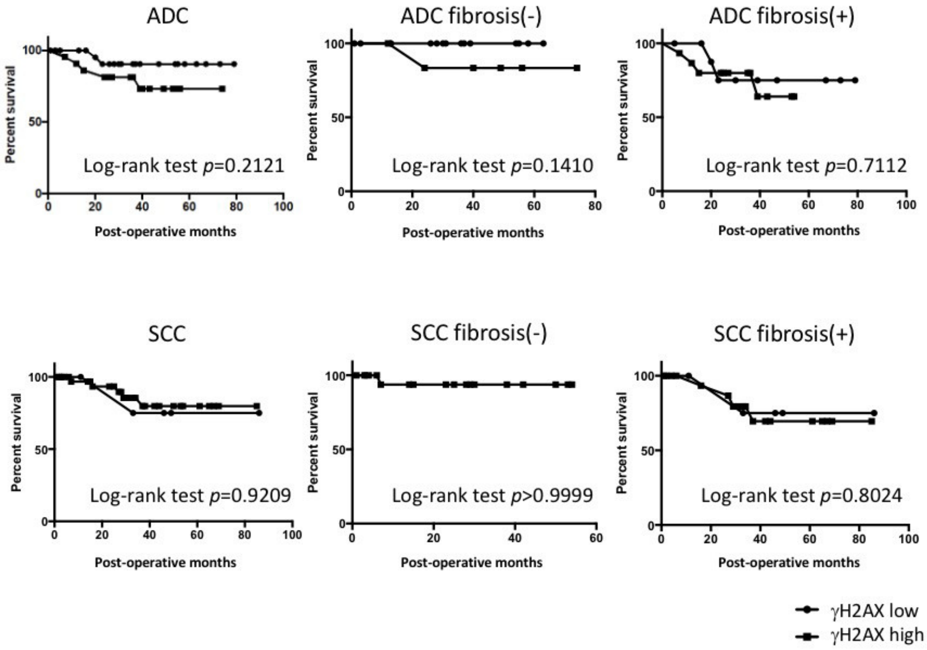

Figure S1. γH2AX expression and post-operative survival. No statistical significance in terms of patient survival was detected in high/low γH2AX expression of adenocarcinoma, adenocarcinoma without fibrosis, or adenocarcinoma with fibrosis.

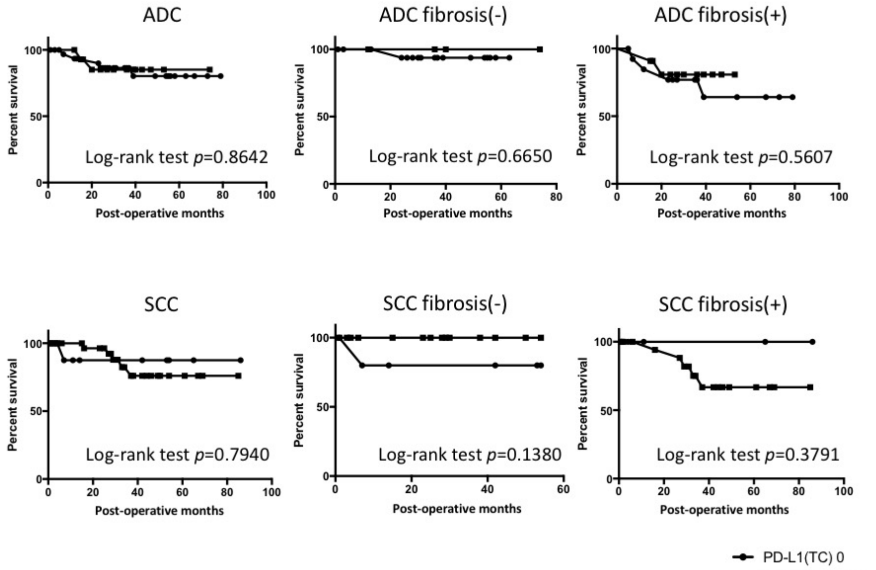

Figure S2. PD-L1 expression and post-operative survival. No statistical significance in terms of patient survival was detected in high/low PD-L1 expression of adenocarcinoma, adenocarcinoma without fibrosis or adenocarcinoma with fibrosis.
